# Protocol: How can people with social care needs be supported through processes of digital care navigation to access remote primary care? A multi-site case study in UK general practice of remote care as the ‘new normal’.

**DOI:** 10.3310/nihropenres.13385.1

**Published:** 2023-04-05

**Authors:** Gemma Hughes, Sarah Rybczynska-Bunt, Sara Shasha'h, Sarah Greene, Sara Shaw, Trisha Greenhalgh

**Affiliations:** 1 Nuffield Department of Primary Care Health Sciences, University of Oxford, Oxford, OX2 6GG, UK; 2 Penisula Medical School (Faculty of Health), University of Plymouth, Plymouth, PL6 8BX, UK

**Keywords:** Care navigation, digital care navigation, primary care, access, qualitative research

## Abstract

**Background:**

Care navigation refers to support for patients accessing primary care and other related services. The expansion of digitally enabled care in the UK since the coronavirus disease 2019 (COVID-19) pandemic has led to a greater need for *digital* care navigation: supporting people to access primary care digitally and, if necessary, to help them find alternative non-digital routes of access. Support to patients with social care needs (including but not limited to those who are homeless and insecurely housed, living in residential care and supported by domiciliary carers) increasingly involves work to navigate primary care provided remotely and accessed digitally. There is little knowledge about how this work is being done.

**Methods:**

*Care Navigation* involves embedded researchers identifying digital care navigation for patients accessing services in 11 GP practices recruited to a linked study of remote primary care ( *Remote care as the ‘new normal?’*). Digital care navigation will be studied through go-along (in-person or remote) interviews with a sample of 20 people offering formal (paid or voluntary) support, 6 national and regional stakeholders who plan, commission or provide digital care navigation and a focus group with 12 social prescribers engaged in digital care navigation. A co-design workshop with people working in, or commissioning, social care settings will consider how findings can inform improved digital care navigation, for example through the development of resources or guidance for care navigators.

**Results (anticipated):**

Findings are anticipated to include evidence of how digital care navigation is practised, the work that is done to support patients in accessing remote primary care, and how this work is shaped by material resources and variations in the configuration of services and infrastructure.

**Conclusions:**

New explanations of the work needed to navigate digital care will inform policy and service developments aimed at helping patients benefit from remote primary care.

## Plain language summary

Since the COVID-19 pandemic, UK general practices have increased the offer of remote care. Patients can book appointments online and via smartphones. People who are homeless or insecurely housed (living in temporary accommodation, hostels and shelters) and those who live in residential care or who have paid carers at home can find it hard to access remote care through digital devices and over the internet, even though it could be convenient and beneficial for them. 

This study, linked to the *Remote Care as the ‘new normal’* study, will find out about the work that is done to help such people ‘navigate’ (or, in other words, to support people to access) remote care. We are interested in the additional work that people in paid roles, such as care home staff and domiciliary carers, might do alongside their main duties to help patients get online. We are also interested in the extra work that social prescribers (who refer patients to services in the community) have to do to support digital access to services.

We will find people who are providing support to patients at the 11 GP practices included in the linked study across England, Wales and Scotland and find out how they do this work, what helps them to do this and which organisations they work with. We will conduct ‘go-along’ interviews (in-person or online) where people show us how they do their work and explain it to us. We will interview people who make national and regional decisions about how to fund and support digital care navigation and hold a focus group with social prescribers to find out what they think about the work that is done to help people access remote primary care.

Based on what we learn, we will make suggestions to improve digital care navigation.

## Introduction

During the coronavirus disease 2019 (COVID-19) pandemic, the use of digital communications changed dramatically, leading to some concerning implications for digital inequalities for people with poor digital access or literacy ( Nguyen *et al.*, 2020). Digital disparities have been widely reported, for example in video consultations, where low household income, older age, and ethnic minority background (especially limited-English speakers) have been associated with lower uptake ( Husain *et al.*, 2022).

UK general practices now routinely offer remote consultations in addition to face-to-face. General practices also offer digital methods of booking remote and in-person consultations, ordering repeat prescriptions and accessing diagnostic results. The expansion of remote care through digital means can be convenient and beneficial for many patients. However, digital inequity can lead to digital health disparities whereby those people who might benefit most are least able to access remote care ( Alami *et al.*, 2022). Increasing digital access therefore has the potential to generate further inequities ( Veinot *et al.* 2018). Additional work is required to support people with poor digital access to benefit from remote care, and so address health inequities.

This study, *Care Navigation*, aims to identify and theorise how such work is done to support people who are likely to have poor digital access to benefit from remote primary care in the UK and, through a process of co-design, make suggestions to improve digital care navigation. *Care Navigation* was commissioned by the National Institute for Health and Care Research to extend *Remote Care as the ‘new normal’*, part of the Remote by Default (RbD) programme (see Box 1) by focusing on social care needs and outcomes and underserved groups. *Care Navigation* will focus on three groups of people who are both at risk of being marginalised by the expansion of technology and internet-enabled primary care and have regular contact with staff for a range of social care support needs: homeless/insecurely housed people, care home residents, and people in receipt of domiciliary care. Informed by findings from RbD, the thematic focus of this study will be on the quality of digital support, access barriers and enablers, workforce and infrastructure (see Box 2).

**Box 1. The Remote by Default study**

The expansion of remote care in the UK during and since the pandemic in 2020 has been studied across 11 general practices by the Remote by Default (RbD) study in two phases. The participating practices are geographically and economically disparate serving different patient cohorts, located across three of the four nations (England, Wales, and Scotland) with seven of the practices situated in areas of moderate or high levels of deprivation.

The first phase, now known as Remote by Default 1 (RbD1), addressed remote care in general practice from June 2020 to November 2021 and explored the rapid expansion of video and other remote forms of consultation driven by the conditions of the pandemic.

RbD1 found that understanding how remote care was accomplished required considering the practical and contextual dynamics of primary care. The Planning and Evaluating Remote Consultation Services (PERCS) framework was developed as an explanatory model of how political, organisational, economic, technical, relational and clinical complexities shape the emerging phenomenon of remote primary care ( Greenhalgh *et al.,* 2021). One domain of this model is digital inclusion. Measures required to improve digital inclusion for remote care include diversity of provision, including non-digital options, and support for digital access.

Findings to date from these, and other related studies, include insights into both the processes of changes to remote care and the consequences of those changes. The spread and scale up of video consultations, for example, can be understood as a form of social change and a changing social practice ( Hughes *et al.*, 2022), related not only to the crisis conditions of the pandemic but shaped by pre-existing infrastructures ( Wherton *et al.*, 2021) and in-pandemic policy changes ( Shaw *et al.*, 2021). Whilst there has been variability in the uptake of video consulting in general practice ( Greenhalgh *et al.*, 2022), a series of consequences have arisen from both the broad policy support for, and increased use of, remote consultations. These include changes to the nature of risk during remote consultation ( Rosen *et al.*, 2022) and wider changes to institutional infrastructures as new routines become embedded ( Wherton *et al*., 2022).

The second phase, Remote by Default 2 (RbD2), studies the challenges of sustaining the changes made to remote care to from 2021 onwards ( Greenhalgh *et al.*, 2022a).

**Box 2. Study themes**

1. QUALITY DIGITAL SUPPORT, best practice components (i.e. time and space) for supporting patients before during and after remote consultation;

2. ACCESS BARRIERS AND ENABLERS relating to resource mobilisation for digital capability and the digital literacy levels and readiness of supporting persons;

3. WORKFORCE, including care navigation training and supervision and the acceptability of digital skills embedded into supporting role;

4. INFRASTRUCTURE, including technical (hard/software, interoperability); standard operating procedures and capacity to offer support for urgent requests for appointments.

Previous empirical work by the study team found that people in supporting roles had to extend that support to help patients navigate online access to healthcare ( Greenhalgh *et al.*, 2022b). Digital disparities, such as ownership of digital devices and access to the internet, were exacerbated by other difficulties in accessing remote care ( Rosen *et al.*, 2022). People who were homeless or insecurely housed, for example, did not have ready access to the appropriate private, quiet, spaces required to conduct a video consultation. Additional support was required, with, for example, the assistance given by voluntary sector staff to homeless people attending a soup-run extending from traditional support with accessing warm food, shelter and healthcare to help navigating online services and finding a suitable location from which to consult remotely. 

## Background

Care navigation generally refers to supporting patients in knowing about, attending and benefiting from services that include medical care, other welfare services and broader community resources that can support health and wellbeing. Care navigation therefore refers to a range of activities that aim to support patients to access primary care, as well as connect to other health and related services from and beyond primary care. It is related to (and at times synonymous with) similar activities such as social prescribing, and is generally understood to be added to existing roles as a set of competencies rather than a specific post. Similarly, *digita*l care navigation or digital support is an additional set of tasks and skills that people working with patients are expected to acquire in order to support them to use and benefit from online services, and online access to services.

The origins of the concept of care navigation can be traced to patient navigation programmes that originated in the US in the 1990s to support poor people requiring cancer care ( Freeman & Rodriguez, 2011). Patient navigation described different kinds of support for poor, uninsured and underinsured people to help them gain access to and through services. The programme was a response to the identified risk of health disparities accruing due to barriers for vulnerable populations to access timely diagnosis and treatment. Navigators were lay people or healthcare providers who supported patients to overcome barriers such as lack of funds, information, trust or communication. 

### Care navigation in UK policy

Care navigation, rather than patient navigation, is the term used in the UK. Care navigation has been associated with three interrelated policy concerns in the UK: primary care capacity and access ( General Practice Forward View, 2016), integration of care for patients with multiple and complex needs ( The NHS Long Term Plan, 2019), and support for people to access non-medical care, which has become known as social prescribing ( Tierney *et al.*, 2019). Care navigation is understood by Health Education England as an interventional process rather than a specific role or post and something that should be part of all staff work ethos and duties. Care navigation, according to Health Education England, can be offered in three tiers (essential, enhanced, expert) according to the complexity of the patient and the skills of the navigator ( *Care Navigation: A Competency Framework*, 2016). Digital care navigation has become a more recent focus of concern ( *Digital inclusion guide for health and social care*, 2019).

The 2016 *General Practice Forward View* (GPFV) set out new investment in primary care in England in recognition of comparative historic underfunding compared to hospital services, which had resulted in increasing workforce pressures and concerns from patients about access to primary care. An earlier policy initiative, the Prime Minister’s Challenge Fund, 2013 (which became known as the GP Access Fund) had provided investment for pilot projects to test out ways of extending or enhancing access to primary care such as extended opening hours and care navigation support. In the GPFV, care navigation was presented as a way of releasing capacity in primary care, with £45 million extra funding available nationally over 5 years for every GP practice to train and support their reception and clerical staff to play a greater role in care navigation. The intention was that these staff would do more patient signposting and handle clinical paperwork, thereby freeing up GP time. 

Supporting patients to navigate through complex systems has been a feature of policies and practices seeking to integrate care for certain patient groups across multiple services and settings. Examples can be found in case studies from Tower Hamlets, where care navigators aimed to prevent A&E attendances and hospital admissions for patients with complex health and social needs and Camden, where care navigation supported patients across the stroke pathway ( Mulimba & Prus, 2016). Such better integrated, or joined-up care, aligns with the concept of more personalised care in the 2019 NHS Long Term Plan ( *The NHS Long Term Plan*, 2019).

In policy terms, care navigation is also used to describe support for people to access non-medical services that are important to people’s health and wellbeing. Various terms are used to describe this work, with a range of activities performed *.* In primary care settings, examples include community link workers in Scotland ( Community Link Worker Initiatives in Primary Care: Key Learning From UK Studies, 2019) who provide welfare advice amongst other forms of support and social prescribers who signpost patients to local support and services. A 2019 survey of English CCGs found 75 different titles used to describe this kind of work (including care navigator, social prescriber, link worker, community connector and sign-poster), which was performed by a range of roles in different organisations (including general practice receptionists and practice managers, specific paid roles and, in one case, volunteers) ( Tierney *et al.*, 2019).

A focus on digital care navigation can be traced from general policy ambitions for increased digitally-enabled primary care, as set out in NHS Long Term Plan (2019) and the Scottish Government’s 2018 Digital health and care strategy ( Scotland's Digital Health and Care Strategy: Enabling, Connecting and Empowering, 2018). Specific policy plans for NHS action on digital inclusion were outlined in 2022 by NHS England ( A plan for digital health and social care, 2022). In practice, digital support is added to existing roles and responsibilities, for example NHS Digital (2019) recommend local practitioners serve as digital champions and the NHS Pathfinder project has demonstrated how digital inclusion can be added to social prescribing ( Digital inclusion guide for health and social care, 2019). Digital support is now recognised as part of care navigation, and The Good Things Foundation (a national charity focused on addressing the digital divide) suggest that digital support is best delivered by trusted people within communities ( Digital Inclusion in Health and Care: Lessons learned from the NHS Widening Digital Participation Programme, 2020).

Whilst the policy emphasis has differed across the nations of the UK (for example, Scotland has prioritised technology enabled care since its eHealth Strategy of 2008–2011, and NHS England has addressed primary care access in the General Practice Forward View, 2016), there is evidence of care navigation in each of the four nations. In Wales, the General Medical Services contract in 2022 requires GPs to provide care navigation for all patients and care navigation training for all patient facing staff ( Guidance for the GMS contract: Access Commitment 2022/23, 2022). Examples of care navigation in Northern Ireland include a training programme for general practice staff and partnerships with the voluntary sector to create community navigators for older people ( Health and Social Care Board, 2019; Age NI, 2022).

### Digital care navigation research

There is little research into digital care navigation, and none that specifically examines digital care navigation for the potentially marginalised groups in the UK that are the focus of this study. There is a body of research on primary care access for marginalised groups, including studies of interventions and experiences of homeless people ( Gunner *et al.*, 2019), refugees and migrants ( Scott *et al.*, 2022) and people living in deprived neighbourhoods in the UK seeking care during the pandemic (Norman *et al.*, 2019). This research includes evaluation of interventions such as outreach services for homeless people ( Clark *et al.*, 2020; Kopanitsa *et al.*, 2023) and theorisation of the burden of non-communicable diseases and access to health care for migrants and refugees in terms of social capital ( Tan *et al.*, 2021). There is also a growing body of research into social prescribing, including link workers’ perspectives on patient engagement in social prescribing ( Wildman *et al.*, 2019) and the adoption of care navigation in primary care in the UK ( Tierney *et al.*, 2019). However, these studies do not specifically address digital or remote access and navigation.

Where remote care has been studied, such as evaluations of the use of telephone triage ( Newbould *et al.*, 2017) and signposting ( Siddiqui *et al.*, 2017), there has been a significant focus on evaluating the effect on managing demand for primary care rather than on access for marginalised groups. There are studies of digital interventions that have identified the potential for improved coordination of care, such as ehealth for older adults living at home people ( Fjellså *et al.*, 2022) and the management of high intensity users of primary care through an online platform (Leung & Qureshi, 2022). Whilst concerns about digitally supporting disadvantaged and vulnerable individuals have been raised by research into social prescribing during the pandemic ( Fixsen *et al.*, 2022), there remains a gap in evidence about how this digital support is practiced. This study will contribute to addressing that gap. 

## Methodology: practices of care navigation

This study considers care navigation as social practice and therefore understands the human actions and judgements that make up the processes of care navigation as being shaped by (and shaping) the context within which they take place ( Shaw *et al.*, 2017). Drawing on concepts of structuration ( Giddens, 1984), studying care navigation as a social practice means analysing the tacit learning, routinized actions, embodied values and behaviours that constitute the work of care navigation. The study will identify the people, activities, actions, events and technologies that constitute and shape the work of care navigation in order to understand how care navigation is performed within the context of increasing provision of remote care, use of online services to book and access care, and concerns about digital disparities. This will involve understanding how support for patients accessing remote care is enacted in practice and how these practices are shaped by different constraints and resources.

Observations of people carrying out the activities that support patients to access remote care and interviews about those activities will identify the resources and conditions that enable these practices such as the technology, competencies, time and space available for care navigation, and how people provide digital support within their existing roles and contractually agreed work. The methodology of narrative networks will be used to analyse the relationships between technologies and organisational processes as people perform the tasks of supporting patients to access remote primary care ( Pentland & Feldman, 2007). Narrative networks offer a way of representing the multiple components and sequences of events that can, and could, be employed to enable access to remote primary care.

## Methods

### Aims, objectives, and research questions

The study has two main aims:

1. To identify and theorise the practices of digital care navigation to support people who are likely to have poor digital access to benefit from remote primary care in the UK.
2. To use the developing theories of care navigation practices to inform the infrastructure support, training and resources needed to enhance digital inclusion for people with social care needs.

The objectives are to explore: 1) how digital support fits (or not) with existing roles concerned with patient access within general practices, and 2) the interplay between contractually agreed care packages, additional digital support and care navigation that is found connected to general practices.

The study aims to answer the research question: *How can people with social care needs be supported, through processes of care navigation, to access primary care remotely?*

### Study design

This is a qualitative multi-site study with a co-design workshop. The study will explore coordination and delivery of digital care navigation, examining how local, patient and service contexts interplay with digital support by pursuing the themes set out in Box 2. Digital care navigation for patients accessing the 11 GP practices recruited to the linked RBD2 study ( Box 1) will be studied through a combination of interviews and observations. Stakeholder perspectives on digital care navigation will, together with findings from the study of practices of digital care navigation, inform a co-design workshop to generate resources and guidance for digital care navigators.

The study focuses on care navigation for three groups that are potentially marginalised by the expansion of remote, technology and internet-enabled, primary care: homeless and insecurely housed people, older adults in receipt of domiciliary care, and people living in care homes. Whilst these people could benefit from remote consulting, as they might struggle to attend in-person appointments, they are also likely to experience digital exclusion and find the health and social care system difficult to navigate. Further, they have regular contact with staff who can potentially be involved in providing them with support to access remote care and thus act as digital care navigators. Embedded researchers in the RbD2 study ( Box 1) will identify such people (e.g. staff of social care providers including care homes, domiciliary care agencies, housing/homelessness services) who support patients to access remote care. We aim to recruit 20 of these care navigators across the 11 practices.

***Go-along interviews.*** Practices of care navigation will be studied through 20 go-along (in-person or remote) interviews with people involved in supporting patients to access remote primary care (care navigators).

The *in-situ* go-along method enables researchers to immerse in the world of people in supporting roles who have been integrating digital support alongside their day-to-day responsibilities ( Carpiano, 2009). The approach will be carried out either synchronously and asynchronously, according to feasibility and preference of each interviewee. Synchronous go-along interviews will involve a researcher joining a care navigator in-person or via a video link at the point when digital support is being provided. Semi-structured interviews with the care navigator will be conducted either on the same day as the observation or as a follow-up. Asynchronous go-along interviews are a virtual adaptation of the go-along interview aimed at eliciting quality multi-sensory familiarity with people’s contexts ( Shareck *et al.*, 2021). These entail care navigators taking photographs, notes and voice memos as they carry out their work to elucidate on the environmental, patient and service contexts. Photographs will, for example, show the room or equipment used rather than the patient as they are supported to connect to a remote consultation. Documents and recordings will then be shared by the care navigator during a virtual research interview and will serve as prompts for discussion with a researcher. Observations and interviews will help to identify what work works well when supporting patients before during and after remote consultations, the technical challenges, and the digital capabilities, confidence and resources of care navigators to digitally support patients,

***Stakeholder interviews.*** Six ‘elite’ stakeholder (in-person or video) interviews will be conducted with people who have strategic knowledge of social care issues in relation to digital health and remote care. The aim is to understand the broader context for care navigation, therefore interviewees will be sampled from stakeholders from key agencies involved in social care and housing/homelessness. The pool of stakeholders will be informed by relationships forged in RbD1 with local and national policy makers (e.g. NHS England and Improvement, Health Education England, Scottish and Welsh governments) and snowballing sampling (asking interviewees to nominate another senior stakeholder). Maximum variety of interviewees will be sought across the areas of policy, regulation, public-private partnerships, financing and reimbursement.

***Focus group.*** The research team will facilitate an online focus group using MS Teams with up to 12 social prescribers to explore their experiences of offering digital support. The focus group will capture a range of experiences and perspectives of offering digital support since the rapid shift to digitalised health and social care services. The group setting will offer the opportunities for social prescribers to compare experiences and perspectives on the extent to which digital support is integral to the social prescribing role and what infrastructure support might be needed to embed this work into existing roles and responsibilities. Focus group participants will be recruited through social prescribing networks that have been established in two geographically and socio-economically disparate sites (Plymouth and Oxford). Participants will be included if they have offered digital support, and will be purposively sampled to target participants who have supported people with social care needs, including those who are homeless/insecurely housed, living in care home or receiving domiciliary care.

***Co-design workshop.*** The research team will set-up, design and facilitate an online co-design workshop with up to 12 people to address the design question: *“what guidance, best practice or training is needed for support staff to offer digital help to patients seeking to access remote primary care?’* Workshop attendees will be recruited from the Expert Advisory Group for the Remote by Default study, the study’s virtual patient group, social care stakeholders, care navigators and social prescribers that have taken part in the study and, where appropriate, snowballing (i.e. ask participants to nominate others). The co-design approach involves engaging with those people who work with the processes of digital care navigation to elaborate on ways in which technology and working practices can be adapted, interpreted and better aligned ( Papoutsi *et al.*, 2021) – in this case to improve access to remote primary care. The workshop will involve participants exploring the findings of the study (for example by being guided by the research team through graphic and narrative depictions of the processes of digital care navigation observed during the study) with the aim of identifying opportunities for improving those processes. Pending the outcome of the workshop, outputs are likely to be targeted at people working in, or commissioning, social care settings and to include guidance at local commissioner level (implementation support needed) and practitioner level (identifying the essential components of care navigation for different patient pathways). 

### Public and patient involvement

Public and patient involvement (PPI) will be facilitated through the External Advisory Group (see Governance section) for the Remote by Default study that includes a lay chair, and through a virtual group that links into the 11 local patient involvement groups in participating practices. The virtual patient group are developing an interrelated piece of work known as ‘digital buddying’ and are developing an infographic on personalised digital support. Digital buddying has involved members of the PPI group supporting digital access with people who have less capability, skills or capacity to engage with technology. The supporting PPI member then shares both their experiences of offering support and the broader perspectives of the person they are buddying. This form of knowledge sharing is especially important in trying to sustainably reach and interact with seldom-heard communities. Our novel approach to PPI means that the group is well placed to critically review the findings of the Care Navigation study and contribute to co-designed outputs.

### Data management and analysis

All formal interviews (go-along, elite) and the focus group will be audio recorded with consent, transcribed and anonymised. Researchers will also make contemporaneous fieldnotes during or immediately after go-along interviews to capture additional observations. Transcripts, audio recordings and fieldnotes will be stored on an encrypted server at the University of Oxford that meets the highest standards of data security and information governance. Facilitator notes, chat comments and summaries of discussions at the co-design workshop will be captured and stored in the same way.

Using thematic analysis, qualitative data will be processed onto NVivo, which allows for easy storage, indexing, categorising and coding of qualitative data across the research team. A broad coding framework will be used as an initial step for familiarisation and data categorisation that will allow for easy identification of data extracts around the themes of the study (see Box 2). The research team will produce narrative summaries of interviews and observations to combine the various data sources to build a rich narrative of the practices of care navigation. Narrative networks (a way of representing and visualising technology in use) will be created as a process of analysing the actions, connections and sequences that constitute practices of care navigation ( Pentland & Feldman, 2007).

## Ethics and dissemination

### Governance

The study will be overseen by the *Remote by Default* external advisory group that has an independent (lay) chair and diverse representation from policy, clinical care, the commercial sector, people with lived experience, and members of patient advocacy groups and regulatory bodies.

### Ethical approval

Approval has been granted from East Midlands—Leicester South Research Ethics Committee and UK Health Research Authority (September 2021, 21/EM/0170) and subsequent amendments. All research participants will give written informed consent in accordance with our ethics protocol.

### Dissemination

The intention is to produce: (i) an empirically-grounded theorisation of how care navigation work is accomplished for remote primary care (ii) a policy briefing aimed at people commissioning and planning care navigation roles and functions (for example Primary Care Networks and Integrated Care Systems) and (iii) through a process of co-production, guidance on supporting digitally excluded groups for people involved in providing care navigation (such as social prescribers, housing support workers and social care providers). Dissemination will be supported by the RbD2 programme to practices and linked organisations, and through ongoing work with national and regional stakeholders and the Expert Advisory Group to further inform and support digital care navigation practices. 

## Study status

As part of the linked study, *Remote Care as the ‘new normal’,* researchers have started to identify care navigation practices and recruit for go-along and stakeholder interviews.

## Conclusion

The study will provide new theoretical explanations of the work needed to navigate digital care, and will inform policy and service developments aimed at helping patients benefit from remote primary care. By using in-person and remote qualitative methods we will illuminate the practices of care navigation and produce and disseminate resources to improve those practices.

## Data Availability

No data are associated with this article.
