# Supplementary material for: Protocol: How can people with social care needs be supported through processes of digital care navigation to access remote primary care? A multi-site case study in UK general practice of remote care as the ‘new normal’
Source: NIHR Open Res. 2023 Apr 5;3:17. [Version 1] doi: 10.3310/nihropenres.13385.1 (PMC10593327; doi:10.3310/nihropenres.13385.1)
Supplement: Peer review history

# Reviewer report: Reviewer response for version 1

**DOI:** 10.3310/nihropenres.14516.r29170

**Published:** 2023-05-15

**Authors:** Emilie Dionne

**Affiliations:** 1 VITAM Research Center on Sustainable Health, Québec City, Canada; 2 Chemin de la Canardière, Universite Laval, Québec City, Québec, Canada

This article presents a proposed study (study protocol) relating to the use of digital care navigation to support the social needs of people in primary care. The article highlights the great challenges that are raised and created by the 'digital turn' in various sectors of society, particularly care, and the risks associated with increasing health and social inequities. This article highlights the relevance as well as care navigation in complex healthcare systems, that are dynamic and change often, and how this is a particularly strong intervention for people with multifaceted needs (multiple care needs). Digital care navigation is a new complex and this study aims to understand it better, ensuring that it is developed with attention for the risks raised by digitalization and inequities, further disparities. 

 The protocol/article is very well written, clear; the review of the literature is exhaustive, well present, and relevant. The authors show a strong and multifaceted understanding and attention for this topic, and the research study proposed is extremely valuable and relevant in today's ages. 

 The methods section is also quite strong, very pertinent and innovative (emphasizing qualitative methods, collaboration, co-design).

 I believe this article, as it stands, is very strong and ready for indexing. I thank the authors and editors for an opportunity to read and review, and look forward to reading more about the study.

Is the study design appropriate for the research question?

Yes

Is the rationale for, and objectives of, the study clearly described?

Yes

Are sufficient details of the methods provided to allow replication by others?

Yes

Are the datasets clearly presented in a useable and accessible format?

Not applicable

Reviewer Expertise:

primary care, sociology of health, sociology of aging, feminist epistemology, qualitative research, art-based research methods

I confirm that I have read this submission and believe that I have an appropriate level of expertise to confirm that it is of an acceptable scientific standard.

# Reviewer report: Reviewer response for version 1

**DOI:** 10.3310/nihropenres.14516.r29168

**Published:** 2023-05-15

**Authors:** Elizabeth Calhoun

**Affiliations:** 1 Graduate College, University of Illinois Chicago, Chicago, Illinois, USA

This paper outlines a protocol for a study of digital care navigation for people with social care needs to access primary care remotely. 

 The study will work with 11 primary care practices in the United Kingdom (UK). Digital care navigation will be studied through go-along interviews with workers either while conducting their work or with pictures, documents, and recordings for context. Additionally, 6 national and regional policy makers, and a focus group with 12 social prescribers. Finally, a co-design workshop will be held to consider how the findings can improve digital care navigation.

 Care navigation in the UK is considered an addition to regular duties rather than a particular position. This paper helps the readers understand that 3 policies support this work, which is something that the US does not have. 

 The findings of this study will be important to the field as the research team is attempting to understand those with housing insecurity, those living in residential care, and those with domiciliary caretakers.

Is the study design appropriate for the research question?

Yes

Is the rationale for, and objectives of, the study clearly described?

Yes

Are sufficient details of the methods provided to allow replication by others?

Yes

Are the datasets clearly presented in a useable and accessible format?

Not applicable

Reviewer Expertise:

Heath services and intervention research with an expertise in patient navigation.

I confirm that I have read this submission and believe that I have an appropriate level of expertise to confirm that it is of an acceptable scientific standard.
